# Continuous on-line glucose measurement by microdialysis in a central vein. A pilot study

**DOI:** 10.1186/cc12713

**Published:** 2013-05-11

**Authors:** Christina Blixt, Olav Rooyackers, Bengt Isaksson, Jan Wernerman

**Affiliations:** 1 Department of Anaesthesia and Intensive Care, Karolinska University Hospital Huddinge, Department of Clinical Science, Intervention and Technology (CLINTEC), Karolinska Institutet, Hälsovägen, Stockholm, Sweden; 2 Division of Surgery, Karolinska University Hospital Huddinge, Department of Clinical Science, Intervention and Technology (CLINTEC), Karolinska Institutet, Hälsovägen, Stockholm, Sweden

**Keywords:** Continuous glucose monitoring, intravenous microdialysis, intensive care, hyperglycaemia, hypoglycaemia, lactate

## Abstract

**Introduction:**

Tight glucose control in the ICU has been proven difficult with an increased risk for hypoglycaemic episodes. Also the variability of glucose may have an impact on morbidity. An accurate and feasible on-line/continuous measurement is therefore desired. In this study a central vein catheter with a microdialysis membrane in combination with an on-line analyzer for continuous monitoring of circulating glucose and lactate by the central route was tested.

**Methods:**

A total of 10 patients scheduled for major upper abdominal surgery were included in this observational prospective study at a university hospital. The patients received an extra central venous catheter with a microdialysis membrane placed in the right jugular vein. Continuous microdialysis measurement proceeded for 20 hours and on-line values were recorded every minute. Reference arterial plasma glucose and blood lactate samples were collected every hour.

**Results:**

Mean microdialysis-glucose during measurements was 9.8 ± 2.4mmol/l.No statistical difference in the readings was seen using a single calibration compared to eighth hour calibration (*P *=0.09; t-test). There was a close agreement between the continuous reading and the reference plasma glucose values with an absolute difference of 0.6+0.8mmol, or 6.8+9.3% and measurements showed high correlation to plasma readings (r = 0.92). Thelimit of agreement was 23.0%(1.94 mmol/l) compared to arterial plasma values with a line of equality close to zero.However, in a Clarke-Error Grid 93.3% of the values are in the A-area,and the remaining part in the B-area.Mean microdialysis-lactate was 1.3 ± 1.1mmol/l. The measurements showed high correlation to the blood readings (r = 0.93).

**Conclusion:**

Continuous on-line microdialysis glucose measurement in a central vein is a potential useful technique for continuous glucose monitoring in critically ill patients, but more improvements and testingare needed.

## Introduction

A positive effect of a tight glucose control in critically ill patients treated in the ICU was postulated a decade ago. The initial study in 2001 demonstrated a remarkable effect on both mortality and morbidity in ICU patients [1]. Later diverging results have been published, andin a large multi-centre trial, Normoglycemia in Intensive Care Evaluation-Survival Using Glucose Algorithm Regulation(NICE-SUGAR), the treatment group had an even higher mortality than the control group [2].

A concern for an increased risk for hypoglycaemia with the intensive insulin treatment has emerged. Up to 18% of the patients subjected to intensive insulin treatment have had episodes of hypoglycaemia [3]. Whether these hypoglycaemic periods have an impact on the worsened outcome is debated. To reduce the risk for hypoglycaemia, different ICUs use different protocols for monitoring glucose levels with some recommending sampling every two hours, which is time, cost and staff consuming[4]. Today the standard routine for glucose measurement in ICU is repeated blood gas analyses. Bedside measurement devices have been demonstrated to differ more than 20% from the laboratory reference value [5]. In addition, recent studies point to a correlation between the variability of glucose and mortality [6-8].

Due to this, the need for a reliable on-line real time measurement of glucose has emerged. Continuous glucose monitoring (CGM) can be potentially instrumental in preventing hypoglycaemia and a high glucose variability[9].Several different techniques have been developed during recent years, either using interstitial or intravascular positioning for measurements[10]. The use of microdialysis as a tool for measuring glucose in the ICU over longer periods of time has been suggested. The subcutaneous devices have been tested in both adults and children admitted in the ICU and results are diverging[11-14].In critical ill patients tissue perfusion can be altered and, therefore, subcutaneous glucose measurement may be affected and may not represent circulating glucose levels accurately[11].

Intravenous microdialysis has previously been studied and proven useful [15]. However, in critically ill patients the peripheral route is not always accessible and a central vein solution might be preferred[16].In addition, a central vein allows for a catheter with a larger membrane surface, which has been shown to improve the agreement to plasma readings.The purpose of this study was to test a central venous catheter with a microdialysis membrane in combination withan on-line analyser and monitor as a principle for CGM. Continuous online measurements were performed onpatients scheduled for major upper abdominal surgery during surgery and postoperative care for 20 hours. For this study a prototype of a commercial product was used.

## Material and methods

Patients (*n *= 10) scheduled for major upper abdominal surgery between October 2009 and December 2010 at Karolinska University Hospital, Huddinge, were enrolled in this prospective study. Inclusion criteria were patients scheduled for surgery involving a central venous access as clinical routine. Exclusion criteria were: 1). Any coagulation abnormality; 2). Planned placement of central vein catheter anywhere else but in the right internal jugular vein; 3). A central vein catheterposition deviating from protocol; 4). No informed consent; 5). Patient under 18 years of age. The study design was reviewed and approved by the regional ethics committee, EPN, in Stockholm. The subjects were informed about the purpose and the nature of the study and the risks involved before giving written informed consent. This study was observational and,consequently,no change in the perioperative care of the patient was made except for the insertion of an extra central vein catheter with the microdialysis membrane. The patients were studied during surgery and at the postoperative ward for a total of 20 hours. None of the patients needed the ICU ward during the study.

Before surgery, the patients received a standard two-lumen central venous catheter and, in addition, a second one-lumen central venous catheter with a microdialysis membrane.Both were placed in the right jugular vein. As a clinical routine, an intravenous glucose infusion was given (25 mg/kg/h; 5% solution) during the whole study period through the distal lumen of the standard central venous catheter. The tip of the central venous catheter with the microdialysis membrane was placed proximal to the glucose infusion lumen to minimize a high false glucose value. A distance more than 3.5 cm between the tips was the target. The catheter placement was documented postoperatively by chest X-ray. The microdialysis catheter had a dialysis membrane length of 40 mm and a diameter of 4 Fr (Eirus SLC, Dipylon Medical AB, Solna, Sweden). The standard central venous catheter was either a two-lumen catheter with a length of 15cm and a diameter of 5 Fr (BD Careflow^™ ^, Becton Dickinson Medical Surgical Systems, Franklin Lakes, NJ, USA), or a three-lumen catheter with a length of 16 cm and a diameter of 12 F (Mahurkar^™ ^, Covidien, Mansfield, MA, USA). Patients routinely received insulin when glucose levels were higher than 11mM.

**Figure 1:**
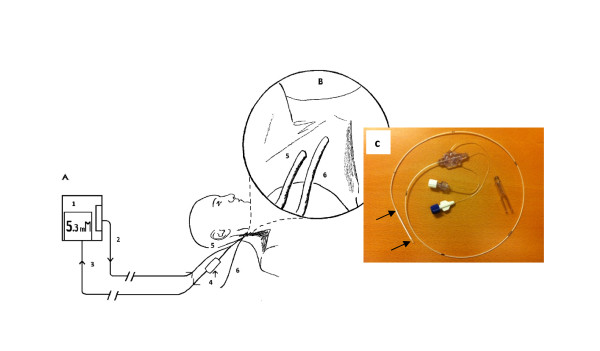
**Schematic design**. A. Schematic design of the microdialysis (MD) system: 1. MD system with sensor holder and display, 2. Perfusate line (sodium chloride), 3. Dialysate line, 4. MD sensor with integrated vials, 5. MD catheter, 6. Adjacent 2 lumen central venous catheter.**B**. Zoomed schematic design of the insertion area; 5. Proximally inserted MD catheter; 6. Distally inserted central venous catheter.**C**.Picture of the central vein catheter with the microdialysis membrane (arrows).

Microdialysis was started immediately after insertion of the catheter but registration started after a run-in time of not less than 30 minutes. Continuous microdialysis measurement then proceeded for 20 hours and on-line values of glucose and lactate were recorded every minute (Eirus, Dipylon Medical AB, Solna, Sweden). The on-line device analyses glucose and lactate every second and the screen present a minute average updated every second. The microdialysis catheter was perfused with saline by a pump located in the Eirus monitor (Figure 1). The microdialysate was analysed for glucose and lactate in an on-line analyser module utilising glucose oxidase (GOD) and lactate oxidase (LOD) followed by an electrochemical detection of H_2_O_2_. Results from the analyser are sent to and displayed on the Eirus monitor (Figure 1). Two separate reference arterial plasma samples were collected every hour, two minutes apart;blood samples were kept on ice and plasma was obtained by centrifugation (1,200xg for 10 minutes at 4^o^C) within 90 minutes andstored at -20^o^C until later analysed. At the same time points, two reference blood lactate samples were collected.These were immediately placed on ice and part of the sample was immediately mixed with 14% perchloric acid to a final concentration of 4.7 mM perchloric and stored at -20^o^C for later analyses. Plasma glucose and whole blood lactate were analyzed as described before[17].Plasma glucose was analysed on an automatic analyser (Konelab 20, Thermo Scientific, Jönköping, Sweden) using a GOD-POD analysis (Thermo Fisher Scientific, Vantaa, Finland). Whole blood lactate was analysed using a 96-well photo spectrometer and a method based on lactate dehydrogenase (DiaSys Diagnostic Systems, Holzheim, Germany).

Retrospectively, the microdialysis measurements were calibrated to plasma glucose and blood lactate values. Two different ways of calibration were used: using the first plasma value only (MD1) or recalibration to plasma glucose every eighth hour (MD8). A mean plasma value of lactate and glucose, derived from the samples collected two minutes apart, was calculated and compared to the microdialysis measurements. The plasma value was compared to the microdialysis value.The transport of the dialysate from the microdialysis catheter to the on-line analysis takes 10 minutes and, therefore, the microdialysis values were corrected for 10 minutes.

Comparisons of the microdialysis and the hourly plasma/blood samples were done using different methods including regression analyses, Bland-Altman plots and Clarkeerror grids. Since this is the first time a central venous catheter with a microdialysis membrane in combination with an on-line sensor was tested, we designed the study as a pilot study and did not perform any power calculations. Student's *t*-test was used for statistical comparison of data.

## Results

Continuous microdialysis proceeded for 20 hours in all patients included in the study. The patients had major upper abdominal surgery; pancreas resection (*n *= 3), liver resection (*n *= 6) or gastric resection (*n *= 1). Ten patients were studied per protocol, eight females and two men, mean age 59.7 yrs (range 27 to 81). Six patients received a BD Careflow^™ ^and four a Mahurkar^™ ^central vein catheter. All patients, at some point during the study, received noradrenalin and five of the patients received insulin. No adverse events were observed that were associated with the central vein catheter or with the microdialysis membrane. The mean distance between the tips of the two catheters was 59mm (range 39 to 82 mm), with the microdialysis catheter as the most proximal. X-ray images were used for documentation of the individual placement.

The time plots of comparisons between all the microdialysis recordings and the hourly blood measurements of all the individual patients can be found in the Additional files. Online microdialysis measurements were presented every minute but only the results from the same time as the hourly blood samples are used for further comparison. A total of 195 individual values were collected and analyzed. The recorded glucose values ranged from 4.2 to 17.1 mmol/l.

Mean microdialysis-glucose using a single calibration (MD1) was 9.6 ± 2.5mmol/l (SD) and eight-hour calibration (MD8) 9.8 ± 2.4 mmol/l (SD). Both calibration methods showed close agreement between the continuous reading and the reference plasma glucose valuesin 8 of the 10 patients(see Additional file 1). MD1 calibration showed a mean absolute glucose difference of 0.85 ± 0.82 mmol/l (SD) or 8.8 ± 8.4% (SD) and MD8 calibration 0.61 ± 0.76 mmol/l (SD), or 6.8 ± 9.3% to the reference plasma value.

**Figure 2:**
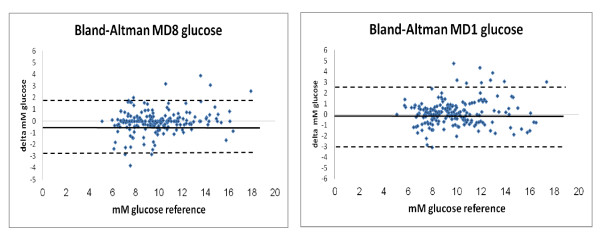
**Bland-Altman plots**. Mean reference plasma glucose vs. microdialysis glucose, eight-hour calibration (MD8; left) and single calibration (MD1; right). Bold line: lines of equality; dotted lines: limits of agreement (1.96*SD).

**Figure 3:**
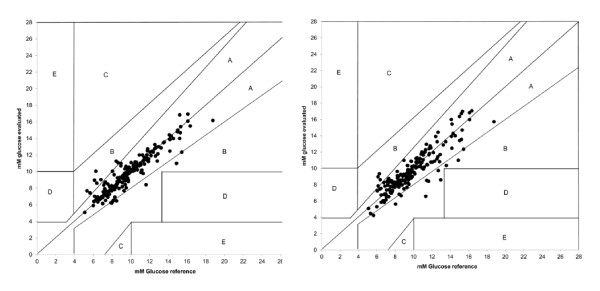
**Clarke-Error Grid**. Reference glucose vs. microdialysis glucose, eight-hour calibration (MD8; left),and single calibration (MD1; right).

Both calibration methods showed high correlations to the plasma readings, MD1 r= 0.89 (*P *<0.001) and MD8 r= 0.92 (*P *<0.001; t-test). The agreement between MD-glucose values and plasma glucose values is presented as a Bland-Altman plot (Figure 2). The lines of equality were close to zero for both ways of calibrating. The MD values showed a limit of agreement ( ± 1.96 SD: CI 95%) of 24.2% (2.34mmol/l) using single calibration (MD1) and 23.0% (1.94 mmol/l) using MD8 compared to arterial plasma levels. The difference in the limit of agreement between the two calibration methods is not statistically significantly different (*P *=0.09; t-test).Presented in a Clarke error grid, 100% of all values are found in the A or B areas. In MD1, 92.7% of the values are in the A-area and 7.3% in the B-area, (Figure 3). In MD8, 93.3% of the values are in the A-area and 6.7% in the B-area.

Lactate measurements of the microdialysis were calibrated in the same way as for glucose, retrospectively using single and eight-hour calibration. Mean microdialysis lactate using either calibration was 1.3 ± 1.1 mmol/l. Although for the lactate the agreement of the microdialysis with the reference value was not as good as for the glucose, the changes over time were very well represented by the microdialysis measurements (see Additional file 2). MD1 calibration showed a mean absolute difference of 0.28 ± 0.35 mmol/l (SD) or 23.4 ± 21.8% (SD) and MD8 calibration 0.25 ± 0.35 mmol/l (SD) or 20.5 ± 22.3% (SD).

**Figure 4:**
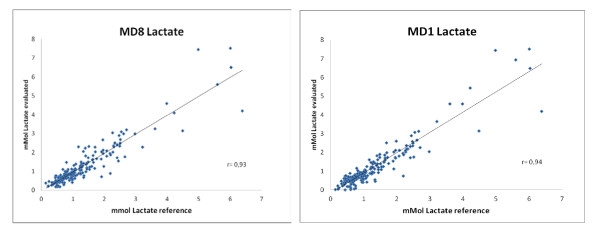
**Regression analysis**. Reference blood lactate vs. microdialysis lactate, eight-hour calibration (MD8; left),and single calibration (MD1; right).

**Figure 5:**
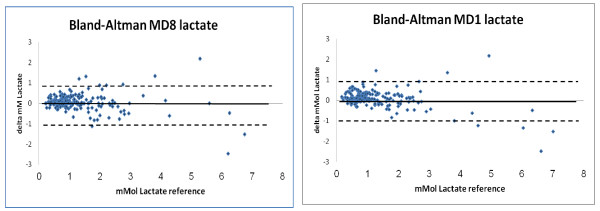
**Bland-Altman plots**. Mean reference blood lactate vs. microdialysis lactate, eight-hour calibration (MD8; left) and single calibration (MD1; right). Bold line: lines of equality; dotted lines: limits of agreement (1.96*SD).

Both calibration methods showed good correlation to the plasma readings, MD1 r= 0.94 (*P *<0,001) and MD8 r= 0.93(*P *<0,001)(Figure 4).The agreement between MD-lactate and blood lactate values is also presented in a Bland-Altman plot (Figure 5).The limits of agreement ( ± 1.96 SD; CI 95%) were for MD1 60.8% or 0.88 mmol/l and forMD860.3% or 0.86 mmol/l (Figure 5).

## Discussion

In the present pilot study, wetested a central vein catheter with a microdialysis membrane in combination with an on-line analyzer and monitor for continuous glucose and lactatemonitoring. Eight of the 10 patients included show good agreement. In the other two patients, the trends in glucose fluctuations were the same, but the absolute agreement was imperfect. Reasons for this are discussed below. The limits of agreement in the Bland-Altman were about 24%. Despite this, all of the values measured with the microdialysis were in the non-dangerous zones in the Clarke-error grid. The lactate correlations were high, but the Bland-Altman plots showed limits of agreement up to 60%.

Our results obtained during major surgery suggest that the central vein monitoring using microdialysis is an attractive approach for patients in need of a central venous line. A central venous approach gave the possibility to use a large enough membrane area for a high flow rate of microdialysis fluid, still with a high level of equilibration. This is impossiblein peripheral veins. Another advantage of the microdialysis techniques is that no blood sampling is needed since the analyses is derived from the dialysate. Using blood sampling may affect the risk for thrombosis and blood stream infections.

Two different calibrations were used, a single calibration (MD1) to the first plasma value and one calibration every eighth hour (MD8). No statistical difference in the results was observed. No apparent drift was observed in any of the patients with either calibration, indicating that a future clinical devise possibly can have limited calibrations.

The results show a good correlation and a line of equality close to zero. However, the limits of agreement were >20% with either the single or every eighth hour calibration protocol. According to the ISO certification criteria for point of care glucometers,glucose values must be within 20% of plasma reference more than 95% of the time, when glucose values >4.1 mmol/l. Below 4.1 mmol/l glucose values need to be within 0.8 mmol/l more than 95% of the time [18].Recently,a consensus meeting concluded acceptable criteria for continuous glucose measurement as 95% of readings must be within 12.5% and99% within 20% of reference standard[19]. According to these upcoming consensus criteria, 85% and 91% of our values were within 12.5% and 20% of the reference standard, respectively. Our results showed that measurements from two patients dominate the outlying values, 69.2% (9/13). If these two patients are excluded from analysis, we reach 95% and 98%, respectively, showing that our approach has the potential to reach these criteria, but that testing in larger studies is needed.Most of the outlier values can be explained by the limitations in our study. The first patientpresented suboptimal recordings despite calibration(see Additional file 1). However, this patient had large changes in glucose levels that were shown by the microdialysis methods but not to the same extent as in the plasma reference values. All these changes were the result of clinical interferences, like changes in glucose infusion, corticosteroid and insulin treatment. The suboptimalagreement may be explained by the differences in arterial (reference sample) and venous glucose (microdialysis measure) level,especially during rapid changes. The ninth patientdemonstrates a possible calibration problem, which might be explained by the fact that we used a prototype analyser. The lactate readings in the same patient showed closer agreement, indicating a functional microdialysis membrane and lactate analyser.The remaining outliers were single "off-line" plasma values in four separate patients.

Despite these outliers, all measurements are found within the A or B area in the Clarke-Error grid. Although the Clarke-Error grid is developed for point-of-care glucose analysers for diabetic patients [20], it may serve as a risk estimator. Similar results were recently obtained in conjunction with cardiac surgery using a similar intravascular microdialysis catheter but the analyses were not performed using the on-line measurements [21].

Our study has several limitations. One limitation in our study is the hourly plasma reference sampling. More frequent samples may have improved the accuracy. In addition, we only measured up to 20 hours and cannot conclude the catheter's performance beyond that point. The study was designed as a pilot study and we included only 10 patients. Larger studies are needed to verify our finding and to study the usefulness of this approach in the ICU.

Another limitation is that we used two separate central vein catheters. To minimize the risk for false high values of glucose at the microdialysis membrane due to the glucose infusion, the mean distance between the two catheter tips was 59 mm. However, postoperative mobilization may have varied the distance between the two catheter tips and at different measurement points this might have varied the influence of the glucose infusion. In the future, a device with an integrated microdialysis membrane to a standard central venous catheter will be available.

In the present study we compared central venous to arterial reference measurements which may also contribute to the disagreement observed. These figures should be compared to plasma glucose analyses sampled at the same time point in an artery and a peripheral vein in stable healthy volunteers, giving a limit of agreement of 12% [16]. However, as mentioned above during fast changes in glucose due to clinical interferences, these two different sites might respond differently.

Finally, since we included surgical patients in this study we only obtained glucose levels between 4.2 and 17.1 mmol/l and not in the hypoglycemic range. Due to this we cannot conclude the usefulness of this approach in the ICU until specifically studied.

Not only glucose measurements are of importance for ICU patients. A high lactate level is often seen in critically ill, unstable patients and is often related to poorer outcome[22]. Therefore, monitoring of lactate could help ICU treatment, especially in unstable situations.An absolute lactate value is of less value than the trend over time in clinical practice.

Lactate analyses have proven sensitive to sample handling and calibration.Lactate concentration is significantly higher in plasma than in erythrocytes[23,24]resulting in a 30 to 40% difference between plasma and whole blood [25].Measurements are, therefore, easily affected if the sample is not immediately stored on ice to minimize the additional lactate production in erythrocytes. In our studies, the lactate was measured in whole blood samples in which all processes were immediately halted by precipitating all the proteins and freezing the sample in liquid nitrogen. Since we calibrated the microdialysis to the whole blood measurements, the difference between whole blood and microdialysate is eliminated. However, we cannot exclude a difference in whole blood and microdialysis lactate appearing over time. In addition, the microdialysis readings were performed in venous blood, whereas the plasma samples were arterial.

## Conclusions

This study has demonstrated that intravascular online microdialysis technique can be a useful tool for continuous glucose measurement. The central venous route is a feasible way of gaining reliable vascular access in ICU patients and it offers the possibility to have a large microdialysis membrane area, improving agreement to plasma values. The use of the combined central vein catheter and on-line analyzer is a potential approach for CGM in the ICU but improvements and more testing, especially in an ICU population, are needed. Further validating studies should use central vein samples as references to eliminate confounders based on these limitations. Lactate values were also analyzed, demonstrating a clear and accurate trend over time but absolute values had a low agreement.

## Key messages

- In this pilot study, glucose measurements by a central venous catheter with a microdialysis membrane combined with an online analyzer recording have been studied in surgical patients over a period of 20 hours.

- The intravascular microdialysis technique shows promising results compared to reference plasma values, justifying further testing.

- The technique may be a useful tool for continuous glucose measurement in ICU-patients, but needs to be tested in this population.

## Abbreviations

CGM: continuous glucose monitoring; CVC: central venous catheter; GOD: glucose oxidase; LOD: lactate oxidase;MD1 or MD8: microdialysis values using single- or either-hour calibration

## Competing interests

The authors declare that they have no competing interests apart from the company providing the catheter and the Eirus microdialysis analyzer.OR and JWreceived a small unrestricted grant and in earlier studies, were the recipients of an unrestricted grant from CMA for performing studies using the intravenous microdialysis technique [16,17]. CMA was the parent company from which Dipylon Medical AB was created.

## Authors' contributions

CB participated in acquiring and analysing data and drafting the manuscript.BI participated in acquiring data and drafting the manuscript.OR and JW participated in designing the study, acquiring and analysing data and drafting the manuscript.All authors read and approved the final manuscript.

## Supplementary Material

Additional file 1 **Diagrams of glucose measurement for each subject**. Diagrams of plasma glucose values vs. microdialysis values, MD8 and MD1 calibration. Click here for file

Additional file 2 **Diagrams of lactate measurement for each subject. **Diagrams of blood lactate values vs. microdialysis values, MD8 and MD1 calibration. Click here for file
