# Supplementary figures and images for: Continuous on-line glucose measurement by microdialysis in a central vein. A pilot study
Source: Crit Care. 2013 May 11;17(3):R87. doi: 10.1186/cc12713 (PMC3707045; doi:10.1186/cc12713)

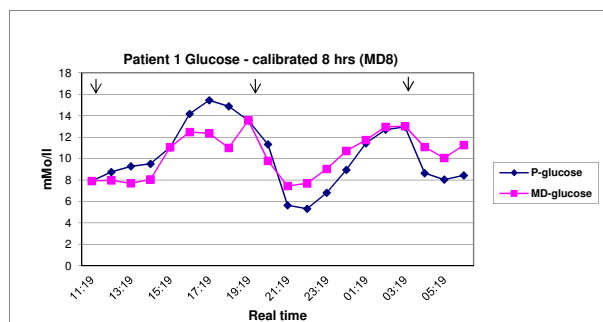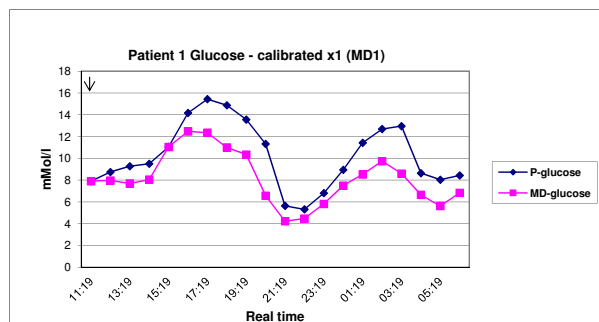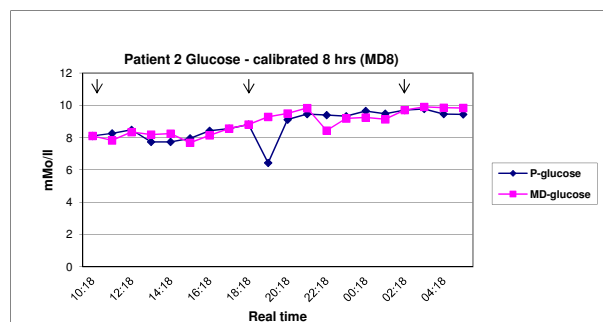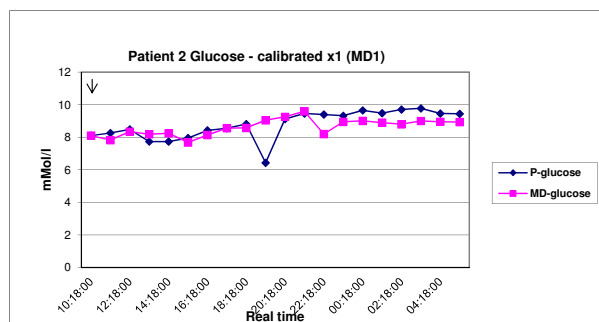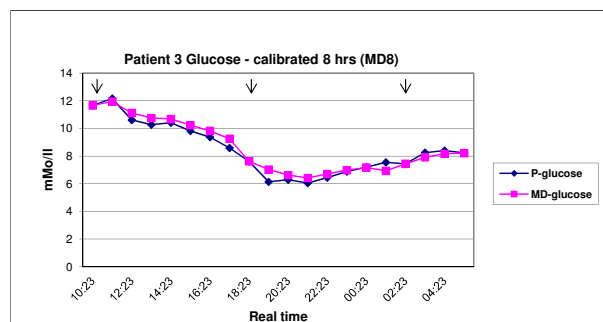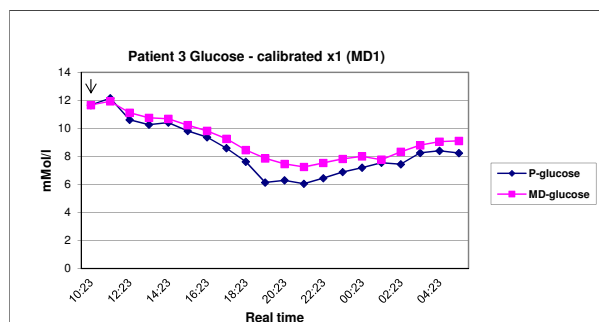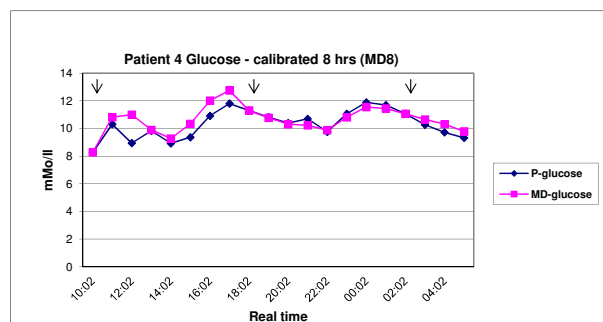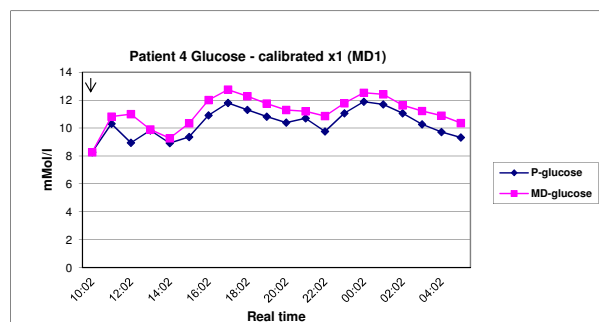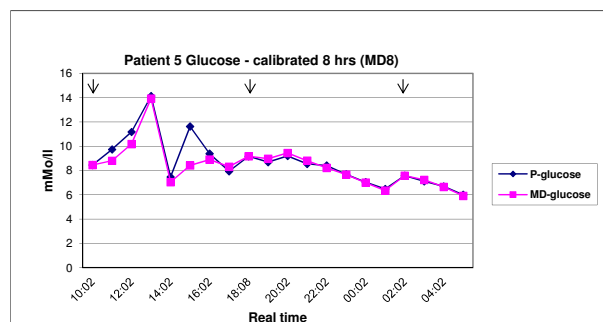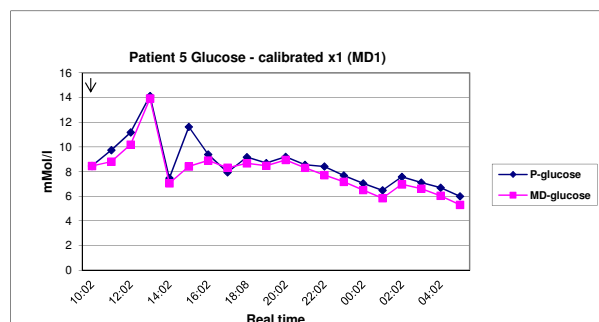

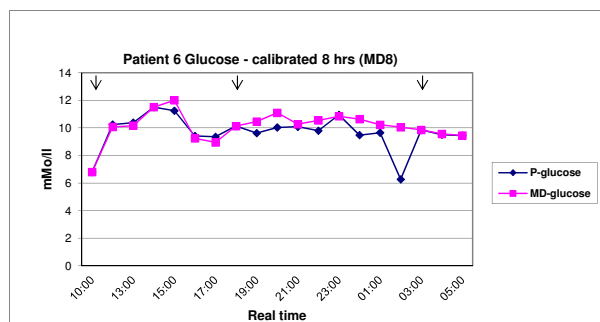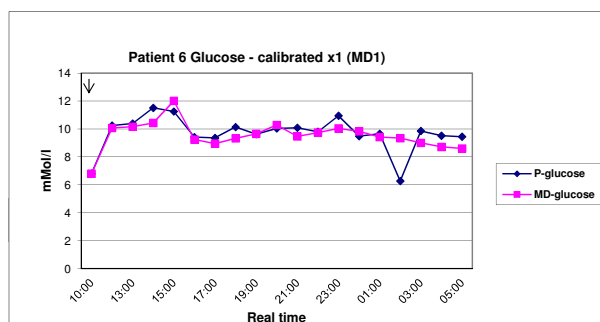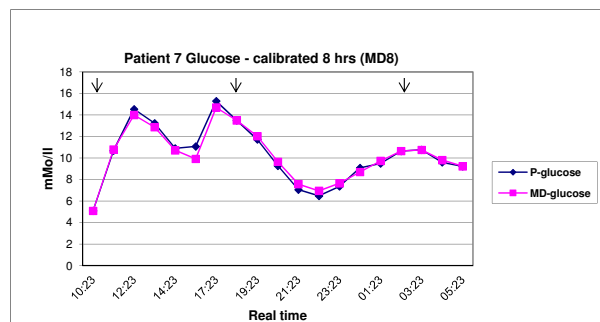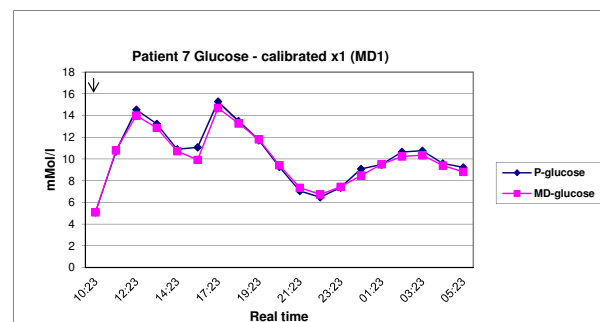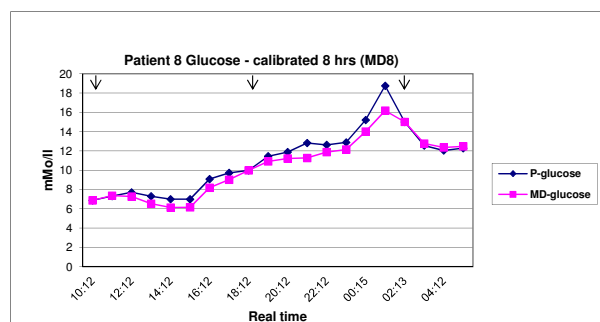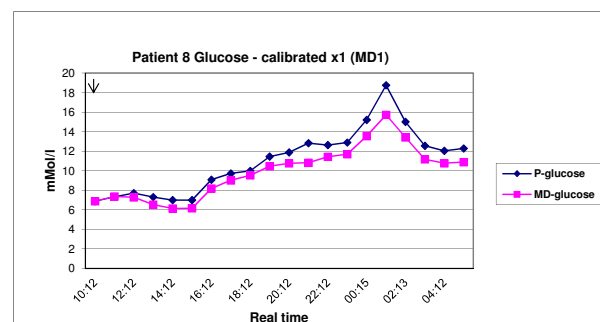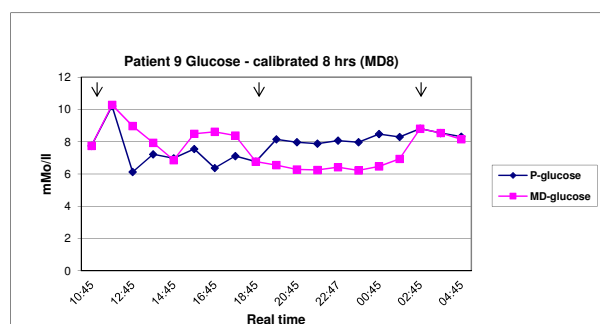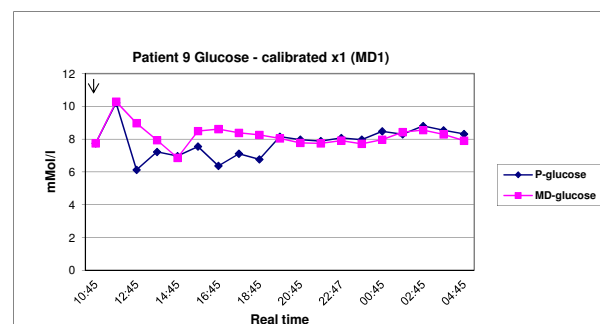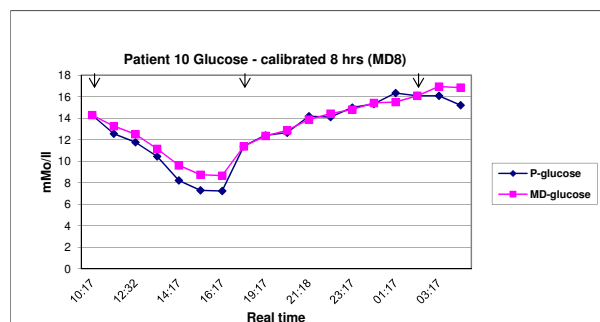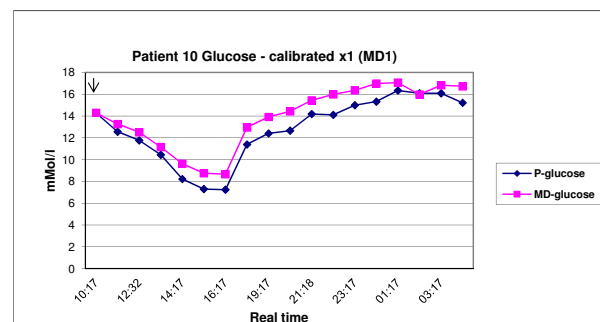

Supplement: Additional file 1 — Diagrams of glucose measurement for each subject. Diagrams of plasma glucose values vs. microdialysis values, MD8 and MD1 calibration. [file cc12713-S1.PDF]

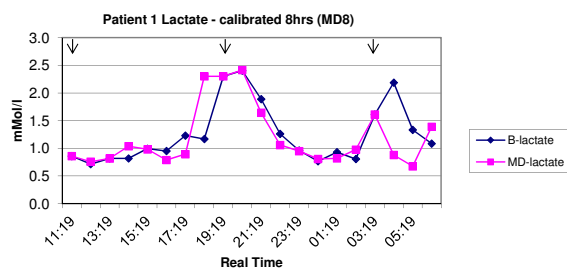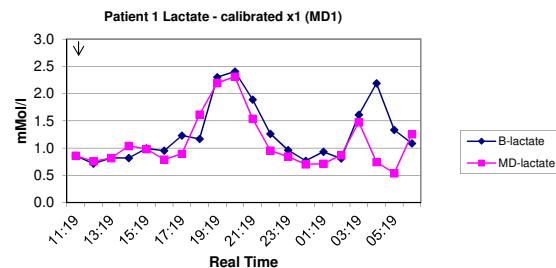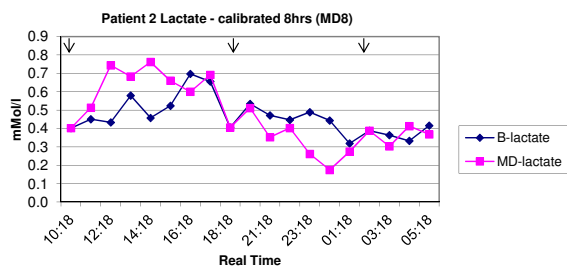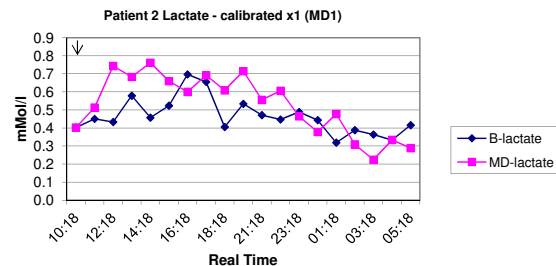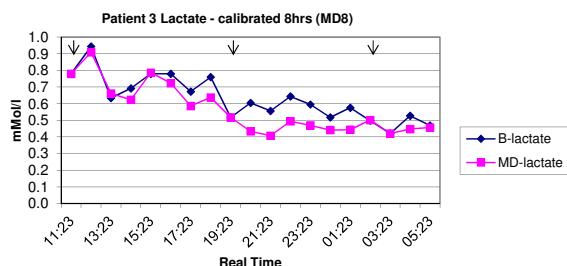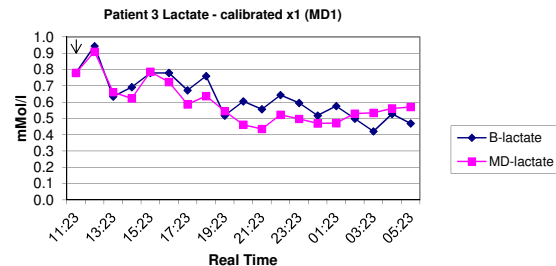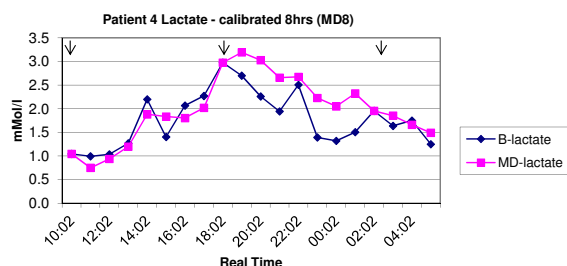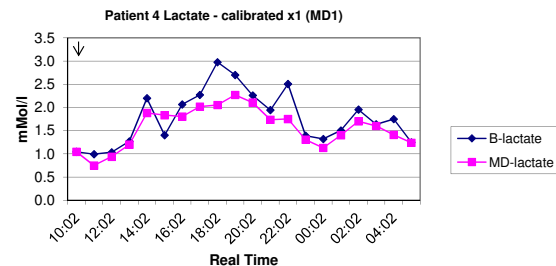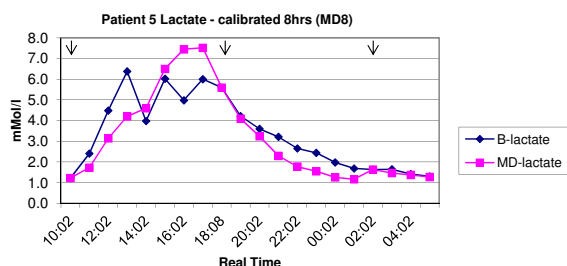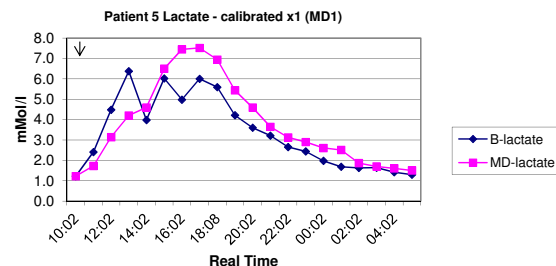

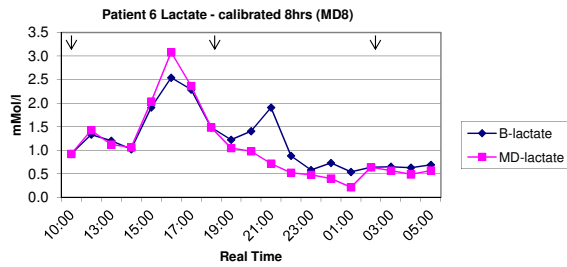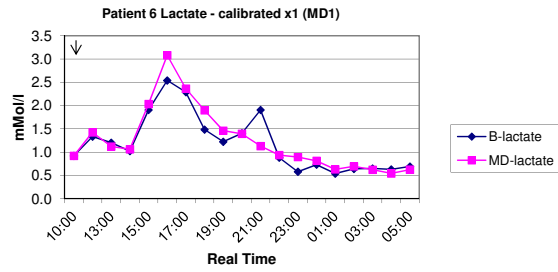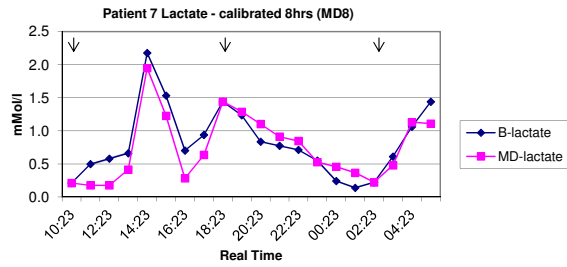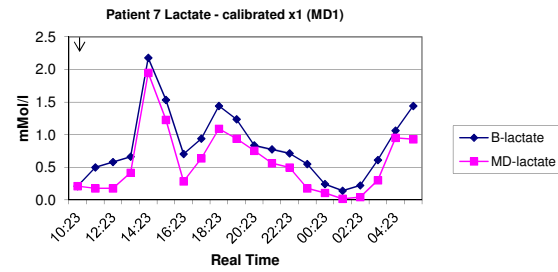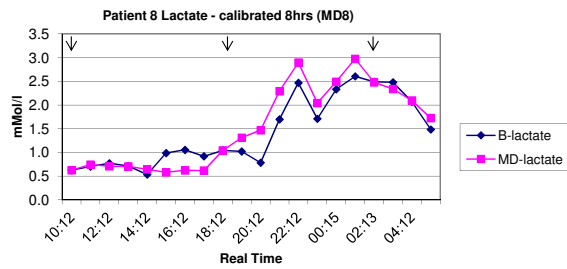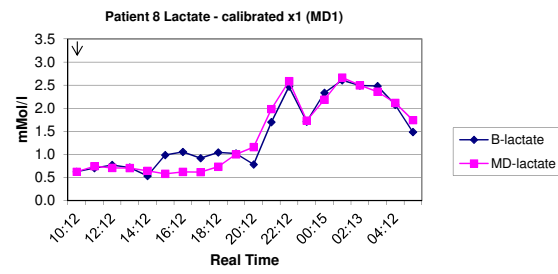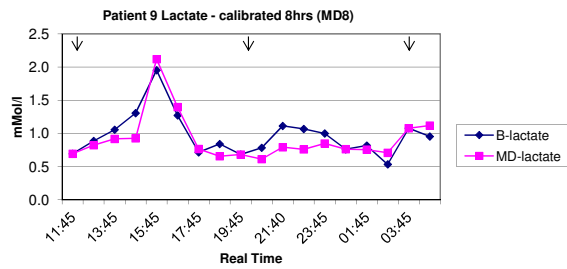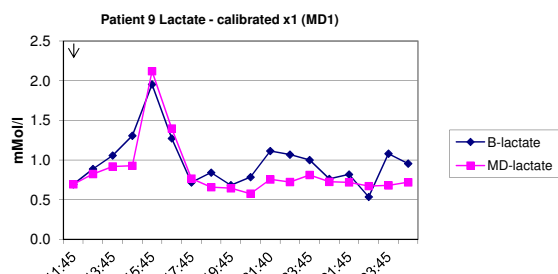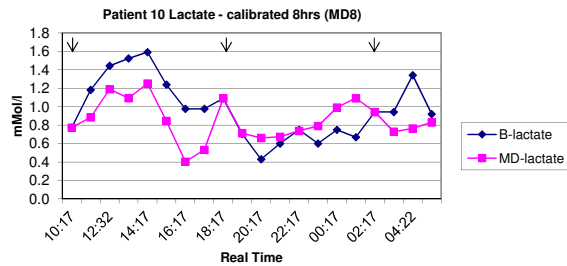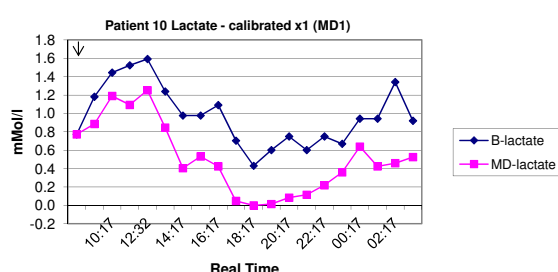

Supplement: Additional file 2 — Diagrams of lactate measurement for each subject. Diagrams of blood lactate values vs. microdialysis values, MD8 and MD1 calibration. [file cc12713-S2.PDF]
